# The role of variable retrieval in effective learning

**DOI:** 10.1073/pnas.2413511121

**Published:** 2024-10-25

**Authors:** Ewa Butowska-Buczyńska, Paulina Kliś, Katarzyna Zawadzka, Maciej Hanczakowski

**Affiliations:** ^a^Faculty of Psychology in Warsaw, SWPS University, Warszawa 03-815, Poland; ^b^Faculty of Psychology and Cognitive Science, Adam Mickiewicz University, Poznań 60-568, Poland

**Keywords:** retrieval practice, spacing, encoding variability

## Abstract

Current research on memory indicates that learning is most effective when it involves spaced retrieval practice of study materials. Here, we investigated whether the benefits of both retrieval practice and spacing can be further boosted when variability is introduced across practice sessions. Across six experiments, participants learned translations of foreign vocabulary, with foreign words embedded in contextual sentences hinting at the meaning of these words. These sentences were then either kept constant or varied from one learning cycle to another. Whenever repeated practice took the form of retrieval from long-term memory with contextual sentences serving as cues (with or without feedback after retrieval attempts), variable sentences led to better learning of the meanings of the embedded foreign words than constant sentences. The benefits of variable retrieval were observed both immediately after study and after a 24-h delay, and they were larger when retrieval practice was spaced rather than massed. However, these benefits were not appreciated by the learners who judged learning to be more effective with constant rather than variable cues. This metacognitive illusion, misaligning the effectiveness of learning and its appraisal by learners, was confirmed in the seventh experiment which focused on learning lecture content. Thus, while spaced retrieval practice employing variable cues clearly produces robust benefits for memory performance, such benefits may be severely underappreciated by the learners.

## Significance

One of the main aims of memory research is to devise strategies that would support effective learning. So far, the golden standard of learning guidelines is supposed to involve repeated retrieval attempts separated in time. Here, we show that the effectiveness of learning via spaced retrieval practice can further be boosted by the addition of another learning technique. We propose that optimal learning conditions can be achieved when learning progresses in a varied (rather than constant) manner: that is, when the same information is processed in a slightly different way at each retrieval attempt, rather than always in the same way. This finding should be taken into account when further developing guidelines for effective learning practices.

One of the main applications of studying memory is to aid actual learning in educational settings. While numerous modulators of memory performance have been described across decades of experimental research on memory, only a handful of them are of use in everyday learning contexts. A comprehensive review of learning techniques (1) has argued that only two such techniques are supported by robust evidence of their effectiveness: engaging in retrieval during learning, termed retrieval practice (2–10), and spacing learning sessions in time (11–20). Thus, the current advice for learning effectively is to attempt retrieval of to-be-learned materials over multiple sessions separated in time (21). Here, we show that the effectiveness of both retrieval practice and spacing can be further boosted. This can be achieved by employing a technique long intuited to improve memory—engaging in variable learning. We demonstrate that the benefits of spaced retrieval practice are particularly strong when variable retrieval is introduced by changing retrieval cues, i.e., prompts for retrieval from memory across learning opportunities.

The idea that encoding variability—learning different facets of some information in each learning session—is crucial for long-term memory retention is an old one (22). The theoretical underpinnings of those putative benefits of variable encoding assign much importance to a defining feature of episodic memory—context, which encompasses all incidental features encoded alongside the to-be-remembered information that can be used as cues to retrieve this information from memory when queried. It has been proposed that with variable encoding, more contextual features become encoded into memory as multiple aspects of to-be-learned information are highlighted (23). Richer contextual representations mean that there is a greater chance that any context present at retrieval would match the context stored alongside the queried information, augmenting the chances of retrieving this information successfully.

While this encoding variability principle is embedded in popular models of memory (24), it is conspicuously absent from evidence-based guidelines for effective learning. One likely cause for this is that historically a number of studies on variable encoding found no firm evidence for its effectiveness in supporting learning, sometimes even documenting better memory under constant rather than varied encoding conditions (25–27). While there are examples of positive effects of variable encoding, they put severe constraints either on the type of encoding processes engaged (28) or on the conditions of retrieval (29, 30), rendering this learning technique too specific to be recommended as a generally useful learning strategy.

Nevertheless, those earlier studies suffer from a key limitation: Most of them focused on repeated encoding understood narrowly as restudying the to-be-learned information. Yet one firm conclusion from memory research is that retrieval practice is often a more effective way of learning than restudy (2). In fact, it has been argued that retrieval from long-term memory is a particularly effective learning strategy because, compared to restudy, it allows for associating the to-be-learned information with richer contextual representations. The episodic context account of retrieval-based learning (31–33) states that during retrieval, episodic context is used as a cue for accessing originally encoded information, which results in the encoding of a novel composite contextual representation, encompassing context features present both at initial encoding and as a cue during retrieval practice. By contrast, during restudy, no intentional contextual cueing takes place, and thus, the restudied information remains associated only with its original encoding context. A richer contextual representation resulting from retrieval practice (as opposed to restudy) may then contribute to the benefits for subsequent memory performance on a delayed test, as originally postulated within the encoding variability theory (23): by increasing the chances of a match of context features present at the time of the final test to those comprising the rich contextual representation resulting from retrieval practice.

If both variable encoding (23) and retrieval practice (31) lead to memory benefits because they allow for creation of rich contextual representations, then the two phenomena should be closely intertwined. Specifically, if retrieval practice, as opposed to restudy practice, ensures creation of composite contextual representations encompassing the original context and the context cue used to tap memory at the practice stage, then the benefits of variable learning should primarily be observed with retrieval rather than restudy practice. Only in the case of retrieval practice should the cues used for variable retrieval be included in the composite contextual representations, ensuring subsequent memory benefits. By the same token, the magnitude of the benefits of spaced retrieval practice should depend on how different the contextual cues utilized across practice sessions are from one another, which should be maximized under conditions of variable retrieval utilizing a variety of cues. In short, thus, the benefits of variability should emerge under conditions of spaced retrieval practice, and at the same time, the benefits of spaced retrieval practice should be maximized under variable learning conditions.

While the idea that the benefits of variable learning could emerge primarily when learning via retrieval rather than restudy practice has been first proposed almost 40 y ago (34), the results of studies testing this idea are far from conclusive. One study that did find clear benefits of variable learning asked participants to engage in solving anagrams, either repeated or changed across practice sessions, which involved retrieval from semantic memory but left the role of retrieval from episodic memory unclear and also precluded a comparison to restudy practice (35). Three further studies that manipulated cue variability across retrieval practice sessions provided diverging results.

In one of these studies (36), participants repeatedly retrieved names when cued with faces accompanied by either constant or changed background photos. In the final test, with no accompanying context photographs, cued-recall performance for names was better when retrieval practice was accompanied by varying rather than constant contexts. Interestingly, in a further experiment, these benefits failed to emerge when retrieval practice was changed to restudy practice. This study, however, while consistent with the theoretical ideas proposed here, induced variability in incidental, unrelated contexts accompanying study. This methodological choice had important consequences. First, it did not provide a good enough match to educational settings, where educators tend to have control over cues/questions used for repeated retrieval, and second, it created a variable condition in which incidental cues—novel on each practice trial—had no way of supporting correct retrieval. As such, the study’s focus is more on costs associated with constant cues rather than the benefits of variable cues. By contrast, a study that specifically looked at varying questions rather than incidental contexts across sessions of retrieval and restudy practice (37) failed to find any difference between constant and varied questions. Here, however, varied questions were only rephrased versions of previously used questions, which could potentially limit the variability necessary to produce memory benefits. Finally, another study (38), varying questions concerning lecture materials, documented benefits of retrieval practice from variable over constant cues, although it also found similar benefits for restudy practice, which differs from the majority of studies on encoding variability (25–27).

Thus, the research conducted to date, although suggestive, does not provide a systematic enough support for the potential role of variable cues in effective learning to recommend this technique as a useful educational strategy. The goal of our study is to provide such support, outlining conditions most conducive for finding benefits of variable cueing. Our first aim in the present study was to demonstrate that imposing variable retrieval by changing cues prompting retrieval of to-be-learned information promotes memory retention of a particular type of educationally relevant materials—foreign language vocabulary. Here, we focused on retrieval cues participants were explicitly asked to use when attempting to retrieve the meanings of the studied foreign words. Our further aims included demonstrating the moderating role of variable retrieval for the benefits of spaced retrieval practice, as well as the assessment of metacognitive aspects of learning via variable retrieval.

## Results

### Experiments 1a and 1b.

In Experiments 1a and 1b, Polish-speaking participants with no previous knowledge of Finnish were asked to learn 40 Finnish words. These words were embedded in sentences presented in participants’ native language, which served as cues for the meanings of the foreign words (e.g., “Dad is sweeping the *lattia”*; the whole sentence, with the exception of the Finnish word, was in Polish, but for readers’ convenience all cueing sentences presented in the manuscript are translated into English). All words were presented across five cycles of practice, and the sentence accompanying each word was either the same across all practice cycles (constant learning condition) or always different (variable learning condition—e.g., “A dog is lying on the *lattia”*; Table 1 for an example). In Experiment 1a, participants were first presented with the whole list of 40 Finnish words and their translations for study. Afterward, they went through five cycles of retrieval attempts for each translation, with sentences and Finnish words serving as cues, but were not provided with any feedback. In Experiment 1b, there was no initial study phase and each retrieval attempt was followed by feedback in the form of the correct translation (e.g., *lattia*–floor). Thus, learning proceeded via retrieval practice in Experiment 1a and via retrieval practice with feedback in Experiment 1b, and this retrieval practice was spaced, with the average lag equal to the number of study pairs. Immediately after the last practice cycle, participants took a cued-recall test in which they were asked to translate the studied foreign words without any sentences provided (e.g., *lattia*–____).

**Table 1.** Example cues across the variable and constant learning conditions in Experiments 1 to 4, translated into English

Learning cycle	Variable learning	Constant learning
Cycle 1	Dad is sweeping the *lattia*.	Dad is sweeping the *lattia*.
Cycle 2	There’s a carpet on the *lattia*.	Dad is sweeping the *lattia*.
Cycle 3	A dog is lying on the *lattia*.	Dad is sweeping the *lattia*.
Cycle 4	Mum is mopping the *lattia*.	Dad is sweeping the *lattia*.
Cycle 5	A cat is sliding on the *lattia*.	Dad is sweeping the *lattia*.

**Fig. 1.**
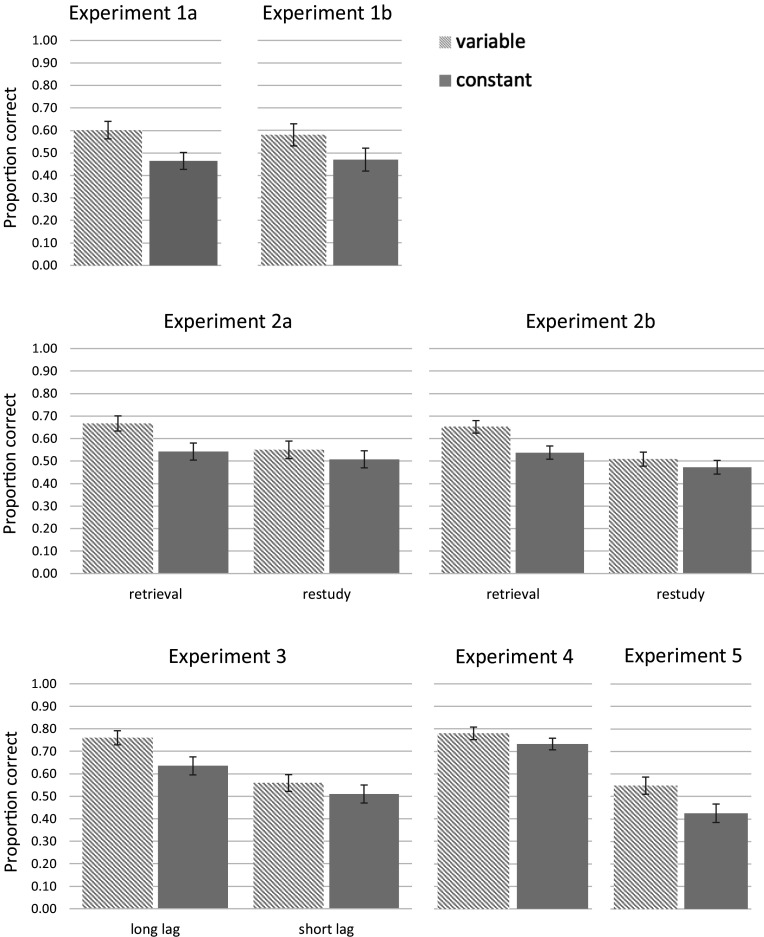
Memory performance on the final test in the variable and constant learning conditions across Experiments 1a to 5. Error bars denote SEM.

For Experiment 1a, the final test (Fig. 1 for the test results for this and the following experiments) revealed better memory performance in the varied than in the constant learning condition, *t* (30) = 4.52, *P* < 0.001, *d* = 0.81. For Experiment 1b, the final test also revealed better memory performance in the varied than in the constant learning condition, *t* (30) = 3.71, *P* < 0.001, *d* = 0.67. These results provide initial support for the idea that the effectiveness of learning via spaced retrieval practice can be boosted when variable rather than constant cues are used for retrieval across learning sessions. They also closely resemble the results of a previous study, which varied contextual cues, in the form of background photos, across repeated retrieval (36). For Experiment 1a, the difference across constant and variable retrieval conditions could stem simply from more exposure to correct responses during learning, given that varied cues provided greater opportunity for deducing the correct answers even if those answers were not successfully encoded in the initial study phase (Table 2 for performance levels during practice across all experiments, which support this contention). However, the use of feedback in Experiment 1b ensured equal exposure to correct answers during practice (albeit at the cost of confounding retrieval practice with augmented learning of feedback following retrieval attempts—a point we revisit in the General Discussion). The similarities across results of Experiments 1a and 1b ensure that the benefits of variable retrieval are robust against variations in procedural aspects of learning.

**Table 2.** Retrieval performance across practice cycles 1 to 5 in Experiments 1a to 4, and cycles 1 to 3 in Experiment 5, in conditions involving spaced retrieval practice

Experiment and condition	Cycle 1	Cycle 2	Cycle 3	Cycle 4	Cycle 5
Experiment 1a					
Constant	0.54 (0.03)	0.60 (0.03)	0.65 (0.03)	0.66 (0.03)	0.68 (0.03)
Varied	0.56 (0.03)	0.68 (0.02)	0.66 (0.03)	0.69 (0.03)	0.75 (0.03)
Experiment 1b					
Constant	0.35 (0.02)	0.83 (0.03)	0.92 (0.02)	0.95 (0.03)	0.97 (0.02)
Varied	0.33 (0.02)	0.64 (0.03)	0.72 (0.03)	0.73 (0.03)	0.86 (0.03)
Experiment 2a					
Constant	0.37 (0.03)	0.86 (0.02)	0.94 (0.01)	0.94 (0.02)	0.97 (0.01)
Varied	0.34 (0.03)	0.73 (0.02)	0.74 (0.03)	0.80 (0.03)	0.90 (0.02)
Experiment 2b					
Constant	0.34 (0.02)	0.87 (0.02)	0.95 (0.02)	0.96 (0.01)	0.97 (0.01)
Varied	0.34 (0.02)	0.73 (0.02)	0.75 (0.03)	0.79 (0.02)	0.92 (0.01)
Experiment 3					
Constant	0.40 (0.04)	0.85 (0.02)	0.93 (0.01)	0.96 (0.01)	0.98 (0.01)
Varied	0.40 (0.04)	0.70 (0.02)	0.76 (0.02)	0.81 (0.02)	0.89 (0.01)
Experiment 4					
Constant	0.36 (0.02)	0.87 (0.02)	0.95 (0.01)	0.97 (0.01)	0.98 (0.02)
Varied	0.34 (0.02)	0.70 (0.02)	0.78 (0.02)	0.82 (0.02)	0.89 (0.02)
Experiment 5					
Constant	0.35 (0.04)	0.68 (0.04)	0.69 (0.04)	–	–
Varied	0.34 (0.04)	0.47 (0.04)	0.46 (0.05)	–	–

Note: SE are given in parentheses.

The manipulation of learning condition was implemented from cycle 2 onward.

### Experiments 2a and 2b.

Experiments 2a and 2b directly assessed whether variable encoding modulates the benefits of retrieval practice to memory performance. For this purpose, variable and constant cues were again compared, but this time for half of the foreign words their meanings were provided outright for restudy, resulting in a 2 (learning condition: varied vs. constant) × 2 (learning mode: retrieval practice vs. restudy) within-participants design. This again follows in the footsteps of a previous study on variable retrieval using incidental contexts as cues (36), but it contrasts retrieval and restudy practice directly. In Experiment 2a, the test was administered immediately after the last study cycle. However, the benefits of retrieval practice—compared to restudy—are often observed only with a delay between learning and the final test (2, 39, 40). Thus, in Experiment 2b, a 24-h delay was introduced to assess whether variable retrieval modulates the benefits of retrieval practice when they are most likely to emerge. Both experiments used the practice-with-feedback procedure of Experiment 1b to ensure equal exposure to correct answers during practice.

For Experiment 2a, a 2 (learning: varied, constant) × 2 (learning mode: retrieval practice, restudy) repeated-measures ANOVA performed on the results of the final cued-recall test revealed a significant main effect of learning condition, *F*(1,51) = 15.51, *P* < 0.001, η_p_^2^ = 0.233, with performance being higher in the varied than in the constant condition (*M* = 0.61, *SE* = 0.034, and *M* = 0.53, *SE* = 0.034, respectively), and a significant main effect of learning mode, *F*(1,51) = 11.28, *P* < 0.001, η_p_^2^ = 0.181, with performance being higher when learning involved retrieval practice rather than restudy (*M* = 0.61, *SE* = 0.035, and *M* = 0.53, *SE* = 0.035, respectively). These main effects were qualified by a significant interaction, *F*(1,51) = 5.87, *P* = 0.019, η_p_^2^ = 0.103. In the constant learning condition, there was no significant benefit of retrieval practice over reading, *t*(51) = 1.41, *P* = 0.17, *d* = 0.20. In contrast to that, when learning was varied, retrieval practice led to better memory performance than reading, *t*(51) = 3.60, *P* < 0.001, *d* = 0.50.

The results of Experiment 2b revealed a similar story. A 2 (learning: varied, constant) × 2 (learning mode: retrieval practice, restudy) repeated-measures ANOVA performed on the results of the final cued-recall test revealed a significant main effect of learning condition, *F*(1,68) = 25.30, *P* < 0.001, η_p_^2^ = 0.271, with performance being higher in the varied than in the constant condition (*M* = 0.58, *SE* = 0.026, and *M* = 0.51, *SE* = 0.026, respectively), and a significant main effect of learning mode, *F*(1,68) = 18.42, *P* < 0.001, η_p_^2^ = 0.213, with performance being higher when learning involved retrieval practice than restudy (*M* = 0.60, *SE* = 0.027, and *M* = 0.49, *SE* = 0.027, respectively). These main effects were qualified by a significant interaction, *F*(1,68) = 5.71, *P* = 0.026, η_p_^2^ = 0.071. This time, in both learning conditions retrieval practice benefited performance compared to reading, although those benefits were more pronounced in the varied condition, *t*(68) = 5.05, *P* < 0.001, *d* = 0.61, compared to the constant condition, *t*(68) = 2.10, *P* = 0.040, *d* = 0.25. Thus, taken together, both experiments demonstrate that the benefits of retrieval practice are more robust when learning is variable, leading to clear effects of retrieval practice even under conditions of immediate testing—where such effects are less consistently present—and magnifying these benefits after a delay. Thus, the effects of two strategies that aim to aid learning—retrieval practice and varying contextual information—can be superadditive.

### Experiment 3.

Experiment 3 assessed whether variable learning modulates the benefits of spaced retrieval practice. For this purpose, one group of participants performed exactly the same task as in Experiment 1b, with an average lag of 40 items between repetitions of the same foreign word. Another group of participants was presented with mini-blocks of two foreign words with their respective sentences—one in the constant and one in the varied learning condition—that they also learned via retrieval practice with feedback, resulting in an average lag of 0.5 item. The final test took place right after the final practice session.

A 2 (learning: varied, constant) × 2 (lag: short, long) mixed ANOVA performed on the results of the final cued-recall test revealed a significant main effect of learning condition, *F*(1,78) = 39.39, *P* < 0.001, η_p_^2^ = 0.336, with performance being higher in the varied than in the constant condition (*M* = 0.65, *SE* = 0.026, and *M* = 0.57, *SE* = 0.026, respectively), and a significant main effect of lag, *F*(1,78) = 9.97, *P* = 0.002, η_p_^2^ = 0.113, with performance being higher when the lag was longer rather than shorter (*M* = 0.69, *SE* = 0.035, and *M* = 0.54, *SE* = 0.035, respectively). These main effects were qualified by a significant interaction, *F*(1,78) = 6.65, *P* = 0.012, η_p_^2^ = 0.079. In both learning conditions, longer lags benefited performance compared to shorter lags, although those benefits were more pronounced in the varied learning condition, *t*(78) = 3.89, *P* < 0.001, *d* = 0.87, compared to the constant learning condition, *t*(78) = 2.31, *P* = 0.024, *d* = 0.52. Thus, just as in the case of retrieval practice, varying cues across retrieval practice sessions modulated the benefits of spacing, which were further boosted by variable learning, providing another example of superadditivity of strategies designed to maximize learning.

### Experiment 4.

The results of Experiments 1a to 3 reveal the benefits of variable encoding when learning via spaced retrieval practice. From an applied perspective, however, it is not only vital that a certain learning technique benefits memory retention, but also that people appreciate this technique as such. This metacognitive insight into the effectiveness of learning techniques may often be lacking, as in the case of retrieval practice (41). This may undermine the effectiveness of learning techniques, making their implementation less likely (42). The question is thus whether people appreciate the effectiveness of introducing variability into learning via spaced retrieval practice.

Experiment 4 assessed participants’ perception of learning via spaced retrieval practice with varied cues. For this purpose, the core design of Experiment 1b was again used, but now it was supplemented with metacognitive judgments measuring participants’ appraisals of the effectiveness of their learning (43). These were collected in the form of a) global predictions that preceded the study phase and reflected participants’ pre-experimental beliefs before any experience with the task, b) item-by-item judgments of learning (JOLs) collected on the final, fifth cycle of practice, immediately after the presentation of feedback, reflecting participants’ appraisal of how well that particular word had been learned, and c) global postdictions following the final test, which reflected participants’ beliefs after experiencing both the constant and variable learning techniques.

The final cued-recall results of Experiment 4 replicated the benefit of spaced retrieval practice with varied cues, *t*(39) = 2.11, *P* = 0.041, *d* = 0.33. Global predictions (Table 3) showed that participants had no preference for learning with varied or constant cues before they experienced the task, *t*(33) = 0.50, *P* = 0.62, *d* = 0.09 (degrees of freedom differ across the analyses as some participants failed to provide one or more interpretable judgment). However, JOLs showed that after the learning phase, they believed that items learned with constant cues would result in better final memory performance than items learned with varied cues, *t*(38) = 3.80, *P* < 0.001, *d* = 0.61. Most importantly, global postdictions revealed that this erroneous appraisal of the relative efficacy of the two learning techniques was still present even after the final test, *t*(31) = 4.16, *P* < 0.001, *d* = 0.73. Thus, while people may not have strong beliefs about the cues they should use for study, they develop such beliefs when they experience both techniques. Unfortunately, in this case, their appraisals become misaligned with the actual effectiveness of learning: They mistakenly believe that spaced retrieval practice is more effective when cues are constant rather than varied.

**Table 3.** Metacognitive judgments in the varied and constant learning conditions of Experiment 4

Condition	Prediction	JOLs	Postdiction
Varied learning	62.21 (2.62)	67.45 (2.56)	56.97 (3.07)
Constant learning	63.82 (2.96)	73.62 (2.5)	67.67 (3.38)

Note: SE are given in parentheses.

### Experiment 5.

All experiments presented thus far used foreign language translations as study materials. While allowing strict experimental control and being of some educational relevance, those materials are not representative of most of the knowledge typically acquired in educational settings. Thus, in the last experiment, we once again aimed at demonstrating the usefulness of retrieval with varied rather than constant cues, this time using materials more typical of learning situations. We also used this opportunity to further demonstrate a metacognitive error associated with variable retrieval practice. Using the materials developed for an earlier study of variable learning (38), we presented our English-speaking participants with five short segments of lectures on geological science. Each segment, covering two or three main concepts, was followed by a series of three questions for each of those concepts. Those questions could either be repeated or varied and were always followed by feedback in the form of a correct response. After studying all five segments and responding to all questions for these segments, a final test followed in which novel, transfer questions were asked, tapping the concepts covered by the lecture and practice questions (Table 4 for example questions). To replicate and extend the metacognitive findings from the present Experiment 4, just before and after the final test, participants were asked to explicitly decide which, if any, learning method—with constant or varied questions—was more effective.

**Table 4.** Example questions targeting the same concept (rift zones vs. subduction zones) in Experiment 5

Question A	An article in a major news magazine alternately describes the Gakkel Ridge, which is located in the Arctic Ocean, as the “slowest known diverging plate boundary on Earth” and a “subduction zone.” Why is this description of the Gakkel Ridge incorrect?
Question B	Subduction zones commonly occur in many different locations on Earth. In contrast, obduction zones are very rare because of the relative densities of the continental and oceanic lithospheres. Obduction zones are similar to subduction zones, but with one key difference. What is an obduction zone?
Question C	A marriage counselor likes to make the analogy to plate tectonics when trying to identify the locus of the problems that couples are experiencing. How is the locus of the marital problems different for “rift” couples and “subduction” couples?
Question D	Despite continuous tectonic activity in the lithosphere, the surface area of the Earth stays constant. Based on this observation, some geologists view plate tectonics as a mechanism for recycling the Earth’s crust. How do rift and subduction zones help to recycle the Earth’s crust?

In the varied condition, three of these questions were used for study, and one question was used for study in the constant condition. In both conditions, a question not presented before was used as a transfer question at test.

The final test results revealed better performance for the transfer questions when practice took the form of answering varied rather than constant questions, *t*(37) = 3.30, *P* = 0.002, *d* = 0.53, replicating the previous results obtained with the same materials (38) and demonstrating once again the benefits of variable retrieval practice. The explicit judgments concerning the effectiveness of practice with constant and variable questions were not distributed randomly either before or after the final test, both χ^2(2, 38)^ = 6.52, *P* = 0.038. This was because the majority of participants judged learning to be most effective with constant questions (both *N*s = 20), with the remaining respondents opting either for varied questions (*N* = 8 before the final test and *N* = 10 after the test) or no difference across the two (*N* = 10 before the final test and *N* = 8 after the test). Thus, participants’ judgments of how effective different learning strategies had been became dissociated from their actual learning by the time of the final memory test.

## Discussion

Across seven experiments, we have demonstrated the benefits of engaging in spaced retrieval practice with varied cues. In this way, the current work serves to firmly establish variable retrieval as a learning technique of fundamental importance. The study extends previous efforts in documenting how encoding variability benefits memory (22–29, 38, 44) by showing that encoding needs to be construed broadly—not necessarily as reading information, but also as a result of retrieving it from memory. Thus, the principle of variable encoding remains intertwined with other highly effective learning techniques: The benefits of variable learning are most readily observed when learning proceeds via spaced retrieval practice. This retrieval specificity of variable learning is consistent with the premise that the benefits of variable learning are underpinned by variability in terms of contextual features, and that contextual features are particularly effectively encoded into memory when they serve as cues for retrieval from long-term memory (31).

Research on retrieval effects in learning has underscored the importance of challenging rather than easy retrieval practice (45–48). Within the episodic context account of retrieval practice benefits (31), retrieval is challenging when it increases the need for reliance on episodic context cues due to impoverished cues embedded in the memory question. In previous research, more challenging retrieval resulted either from withholding part of the cues—when, for example, cued recall was administered instead of recognition (46)—or by using cues weakly rather than strongly associated with to-be-remembered information (45). The results of the present study can also be understood from this perspective of retrieval practice difficulty, with the manipulation of varying cues being another way of instantiating challenging retrieval conditions, necessitating greater reliance on information embedded in the cues, and—in turn—leading to better encoding of this contextual information. After all, repeated retrieval with the same cue should be relatively easy due to well-known regularities of memory such as the principle of encoding specificity (49), which does not contribute to retrieval practice performance when cues are varied.

This understanding of variable retrieval as more challenging for the learner than constant retrieval brings the discussed strategy into the purview of the desirable difficulties framework (50). However, previous investigations of a simultaneous use of learning strategies that come under the umbrella term of desirable difficulties have often found the benefits of combining those strategies to be subadditive (51). For example, it has been found that encoding variability may benefit memory when learning is massed, but undermine it when learning is spaced (26, 52), despite the fact that both variability and spacing are thought to benefit learning by imposing greater challenges for the learner. This contrasts directly with superadditivity of variability and spacing of retrieval practice observed here in Experiment 3, and also conceptually with superadditivity of variability and retrieval practice observed in Experiments 2a and 2b.

We propose that the key to observing sub- versus superadditivity of effective learning strategies is whether learning engages spontaneous or deliberate retrieval. When learning does not involve deliberate retrieval, one of the main determinants of the effectiveness of learning is the occurrence of spontaneous retrieval, often referred to as reminding (53). The effectiveness of reminding, however, depends crucially on the consistency of contextual cues across learning opportunities (54). Thus, when no deliberate retrieval is required, introducing variability in terms of cues undermines the chances of successful reminding compared to keeping the cues constant across repetitions, leading to subadditivity of learning strategies that rest on these two processes. By contrast, when learning involves deliberate retrieval, superadditivity may occur because retrieval is also aided by self-generated cues created during previous study opportunities. The additional use of self-generated cues—deliberate attempts to reinstate previous encoding contexts—can mean that either participants are more likely to retrieve previous study trials for a particular item themselves, or are more likely to remember those trials when feedback is presented. Either way, deliberate retrieval attempts increase the chances of updating context representations with varied cues, which may occur less often when learning rests only on spontaneous reminding.

The understanding of variable retrieval as a “desirable difficulty” is borne out by the results of interim tests taken at study (Table 2). These results support the intuition that retrieval practice is more challenging when cues are varied rather than constant. Performance patterns point to less successful retrieval attempts for varied cues in all experiments except for Experiment 1a. In this particular experiment, practice performance for constant cues was undermined because the correct answers were never provided as feedback. This, in turn, favored retrieval success during practice for varied cues, where the meaning of foreign words could be deduced from various contextual sentences. While the remaining experiments equated exposure to correct answers for constant and varied cues, they did so by including feedback, which means that the retrieval effects described here pertain mostly to the effects of retrieval attempt on subsequent encoding of new information. This feature of our design, while difficult to avoid when comparing conditions necessarily differing in retrieval difficulty, may limit our ability to specify the exact theoretical mechanism of the variability effect. However, it has been previously argued that the mechanisms of benefits conferred by retrieval practice are the same when correct information is retrieved from memory, or when a retrieval attempt fails and correct information is provided externally (10). It seems likely that in both cases, retrieval attempts require using current context as a cue and correct information—whether reinstated internally or externally—becomes associated with this novel contextual representation, supporting future performance via the contextual variability mechanism (31–33).

Finally, if—as we argue—varied cues create more challenging practice conditions, impeding retrieval success at this stage of learning, this provides a clear reason for the metacognitive patterns observed in Experiments 4 and 5. People often base their appraisal of learning on their fluency of retrieval (55) and from this perspective it is unsurprising that they believe that constant cues—which lead very early on to successful practice retrieval when feedback is provided—should eventually lead to better performance. Yet here their beliefs are misguided because fluency of retrieval given one set of cues is not predictive of performance when other, random contextual cues are present on the final test. The fact that the illusion persists even after the test—as documented both by global postdictions reported in Experiment 4 and explicit assessments of strategy effectiveness in Experiment 5—is likely to reflect the difficulty of spontaneously attributing different test items to conditions (56). It is worth noting here that metacognitive errors are most consequential when learners need to choose their own learning strategy, as then these errors are likely to lead to suboptimal choices. The examined strategy of variable retrieval is unlikely to be of particular relevance in this case because the learners are unlikely to practice generating multiple questions by themselves. Still, such questions can be designed by the educators, and in this case, the preference of learners could play an important role: When learners perceive a given strategy to be ineffective, educators may be less willing to implement it. Thus, the challenge for future studies is to devise methods of mending this metacognitive illusion and convincing learners that, when learning, variable and spaced retrieval practice is the way to go.

While this article discusses a learning strategy of potential educational value, it is necessary to point out that much work still remains before variable retrieval can be properly established as educationally relevant. Here, we showed how variability interacts with retrieval practice and spacing, but this was done for relatively simplified materials in the form of translations of foreign vocabulary. While, following a previous study (38), we have demonstrated the general usefulness of variable retrieval practice for materials similar to those used in educational settings, the interactions of multiple strategies applied for such materials need to be further established. Future work should thus focus in the first instance on its generalizability across learning conditions closely modeled on everyday educational practice.

## Materials and Methods

### Experiments 1a and 1b.

#### Participants.

Thirty-one participants recruited via Prolific (age range: 19 to 64 y, mean age: 25.9) participated in Experiment 1a for monetary compensation. Thirty-one undergraduate students from the SWPS University (age range: 19 to 49 y, mean age: 25) participated in Experiment 1b in exchange for course credit. The planned sample sizes for both experiments were 30 participants which ensured the power of 0.85 to detect an effect of d_z_ = 0.50. All participants were fluent Polish speakers with no previous knowledge of Finnish, as specified in the recruitment criteria outlined in the study advertisement. A consent form including all relevant information about the study and conditions of participation was incorporated into the experimental procedure and all participants provided informed consent before the start of the study proper. The study was approved by the Department of Psychology Ethics Committee at the SWPS University.

#### Materials and design.

Forty pairs of Finnish-Polish words were used as study materials. Two additional pairs were used for practice.

In the study phase (Experiment 1a), each Finnish word was presented together with its Polish translation. In the practice phase (both Experiments), Finnish words were presented for study embedded within a sentence written in Polish. For each Finnish word, five cue sentences were created. The full list of forty studied words embedded within cue sentences constituted one study cycle, which was then repeated four times. Half of the Finnish words were presented each time with the same sentence, randomly chosen from the set of five sentences created for this word, and half with five different sentences (Table 1).

The study and practice phases were followed by a cued-recall test for the translations of the studied Finnish words. Participants were presented with Finnish words (without any accompanying sentence) as cues and asked to recall the correct translation. The study had two learning conditions (constant and variable) manipulated within participants. The assignment of word pairs to conditions was counterbalanced across participants and the order of presentation at study and at test was randomized.

#### Procedure.

Participants were tested individually online. They were instructed that their task would be to learn Polish translations of Finnish words in preparation for a future test. They were then told that they would encounter those Finnish words in various sentences written in Polish and encouraged to use the sentence context when learning the translations.

In Experiment 1a, participants were told that they would first study each Finnish word together with its Polish translation for 3 s. Each pair was shown once. They were told that after the presentation of all pairs, there would be a practice phase in which all translations would be tested five times. They were told that they would see the previously studied Finnish words embedded in Polish sentences and that their task would be to provide the correct translation for each Finnish word. They would have 10 s to provide the correct translation, after which the procedure would advance to the next trial. They were also told that across cycles of practice, they would sometimes encounter the Finnish word with the same sentence as previously, and sometimes they would encounter it with a new sentence.

Experiment 1b did not include a study phase. Participants were thus informed that they would see each Finnish study word in five cycles of practice. Their task on the first cycle would be to try and guess the meaning of each foreign word within 10 s, and on the remaining four cycles their task would be to recall this meaning within 10 s. After that time, the sentence would disappear and the Finnish word would be presented for 3 s together with its Polish translation. They were also told that across cycles of practice, they would sometimes encounter the Finnish word with the same sentence as previously, and sometimes they would encounter it with a new sentence.

Finally, participants in both experiments were told that after the fifth practice cycle, they would undergo a final cued-recall test for the Finnish words. After the initial instructions, participants underwent a training phase consisting of five learning cycles and a final cued-recall test for two pairs (one from each learning condition). Then, participants completed the study and practice phases (Experiment 1a) or just the practice phase (Experiment 1b), followed by a self-paced cued-recall test on which they were presented with the Finnish words one at a time and asked to type in the correct translation of each word or press ENTER if unable to retrieve it.

### Experiments 2a and 2b.

#### Participants.

Fifty-two undergraduate students from the SWPS university (age range: 18 to 50 y, mean age: 25) participated in Experiment 2a in exchange for course credit. The sample size was increased here to ensure sufficient power of 0.95 to detect an effect of ƞ_p_^2^ = 0.04 (which would require 53 participants). Sixty-nine participants recruited via the Prolific platform (age range: 18 to 43 y, mean age: 21.5) participated in Experiment 2b in exchange for monetary compensation. The sample size was increased here in case of potential data attrition due to delayed test, which nevertheless did not occur. All participants were fluent Polish speakers with no previous knowledge of Finnish.

#### Materials, design, and procedure.

The materials, design, and procedure were similar to those from Experiment 1b, with one key difference. Apart from manipulating learning condition (varied vs. constant), Experiments 2a and 2b additionally introduced restudy trials for half of the Finnish words. On those trials, the correct translations were provided outright below the cue sentence for 13 s so that no retrieval attempt was required. This resulted in a 2 (learning: varied, constant) × 2 (learning mode: retrieval practice, restudy) within-participants design. In Experiment 2a, the test was administered immediately after the study phase, as in Experiments 1a and 1b, while in Experiment 2b the test was administered after a 24-h delay (± 2 h).

### Experiment 3.

#### Participants.

Eighty participants recruited via the Prolific platform (age range: 18 to 53 y, mean age: 21) participated in Experiment 3 in exchange for monetary compensation. The sample size was further increased to ensure sufficient power of 0.95 to detect the effect size of ƞ_p_^2^ = 0.03 (which would require 71 participants). Participants were fluent Polish speakers with no previous knowledge of Finnish.

#### Materials, design, and procedure.

The materials, design, and procedure were taken from Experiment 1b except for one difference. Half of the participants were presented in the same task as in Experiment 1b, and another half were assigned to a new short-lag condition. In this condition, instead of an average delay of 40 items between presentations of any given Finnish word, as in the previous experiments (here, long lag), the studied word pairs were presented in mini-blocks of two. Within each mini-block, one of the studied word pairs was presented five times with the same sentence (in the constant condition), and another one was presented each time with a different sentence (in the varied condition). This resulted in an average lag of 0.5 item across study repetitions. Thus, apart from a within-participant manipulation of learning condition (constant, varied), Experiment 3 additionally introduced a manipulation of lag (short, long), manipulated between participants.

### Experiment 4.

#### Participants.

Forty participants recruited via the Prolific platform (age range: 18 to 47 y, mean: 30.5) participated in Experiment 4 in exchange for monetary compensation. The sample size was set based on the results of Experiment 1b to ensure the detection of the effect of variability, although we slightly oversampled during testing due to predicted participant exclusions which did not materialize. Participants were fluent Polish speakers with no previous knowledge of Finnish.

#### Materials, design, and procedure.

The materials, design, and procedure were again based on those from Experiment 1b, except for one difference. In addition to comparing the actual effectiveness of learning conditions (varied vs. constant) in terms of memory performance, Experiment 4 additionally measured participants’ beliefs concerning the effectiveness of variable and constant learning. First, before the training started, the two learning conditions were described to participants who were then asked to estimate their final test performance (on a 0 to 100% scale) for the constant and varied conditions (*prediction*). The order in which the conditions were assessed was counterbalanced across participants. On the fifth learning cycle, participants were also asked to provide item-by-item *JOLs*: On each trial of this cycle, immediately after the presentation of feedback, participants were asked to assess their chances, on a 0 to 100% scale, of recalling the correct translation on the final test when presented with the Finnish word. Finally, after the final test participants were asked to estimate on a 0 to 100% scale their final performance for the constant and varied conditions (*postdiction*). For each participant, the order of these postdictions was the same as that of predictions formulated before study. All metacognitive judgments were self-paced.

### Experiment 5.

#### Participants.

Forty-seven participants recruited via the Prolific platform (age range 19 to 59 y, mean age: 34.8) participated in Experiment 5 in exchange for monetary compensation. The sample size was chosen based on the results of a previous study using the same materials (38), where the effect of variability was *d* = 0.49. To achieve 0.85 power to detect this effect, the required sample was 40 participants, but we slightly oversampled in order to compensate for potential exclusions. Ultimately nine participants were excluded: seven due to final test performance ≤ 0.08, one due to copying and pasting responses, and one due to likely using AI to generate responses. This gave a final sample of 38 participants. They were fluent English speakers, recruited from English-speaking countries (United Kingdom, Canada, Australia, United States, Ireland).

#### Materials and design.

Five segments of a lecture on geological science were used as study materials. Each of the segments lasted approximately 8 min and covered two to three geological concepts, with a total of 12 study concepts. Each concept was targeted by four questions which required retrieving the relevant information from the lecture and applying it to answer the specific question. Example questions for a single concept are presented in Table 4.

Each of the concepts was studied either in a constant or in a variable manner. This was achieved by assigning odd-numbered concepts to one condition and even-numbered concepts to the other condition, which was counterbalanced across participants. In the constant condition, for each participant, two questions were chosen from among the four targeting the same concept, one of which was assigned to the study phase where it was presented thrice, and the other was to be used on the final test. The choice of questions to be used for a given participant, and the assignment of questions to the study and test phases, was counterbalanced across participants. In the varied condition, three different questions were used to target the same concept, and the assignment of each of the four questions pertaining to a given concept to the first, second, or third practice trial, or the final test was counterbalanced across participants.

#### Procedure.

Participants were tested online. They were instructed that their task would be to watch five clips about geological science and answer a series of practice questions relating to their content. Some questions would be repeated, but they nevertheless had to be answered each time. Participants were also informed that after watching all five clips and answering the practice questions, they would be given a final memory test for the studied concepts. They were told not to use any external sources of information and rely solely on their memory when completing the experiment.

In the study phase, participants were presented with all five segments of the geology lecture, each lasting between 5 and 8 min. The segments were always presented in the same order. After each segment, participants were asked three questions per concept covered in the given segment, spread across three practice phases. The question related to each concept was either repeated across the three phases—participants answered the same question thrice, or varied—three different questions covered the same concept. The questions were presented one at a time and participants were asked to type in their response. The order in which the studied concepts were queried was constant across the three practice phases. The time limit to answer each question was set to 2 min, although participants could move to the next question earlier if they wished. Each question was followed by feedback in the form of the correct answer, which was self-paced.

After each lecture segment and its question set, participants were asked to assess their confidence in understanding the concepts covered by this segment. They were presented with content labels (e.g., “Explosiveness depends on magma consistency”), one per each concept covered, and were asked to provide their ratings on a scale of 0 to 100. We do not report the data from this task, as in retrospect the clarity of this task to participants was unsatisfactory—participants were likely unable to properly match the labels to the practice questions.

After the study phase, participants were asked which—if any—learning method they considered to be superior, by choosing one of three options: Repeated questions, Different questions, or They were equally effective. This judgment was followed by the final memory test. It consisted of 12 novel questions pertaining to the 12 geological concepts covered in the lecture. As all questions here were novel, answering them correctly required a transfer of knowledge. The questions were presented always in the same order from the first to the last concept covered in the study phase. The test was self-paced, with the exception that the maximum time limit per question was 2 min. Immediately after completing the final test, participants were again asked to indicate which, if any, learning method they considered to be more effective, and they could choose from the same options of Repeated questions, Different questions, or They were equally effective. At the end of the experiment, participants were presented with the correct responses to final test questions.

#### Scoring.

Participants’ practice and final test responses were scored by the first author and two independent raters, not otherwise involved in this study. Interrater reliability (full agreement between all raters) was 84.7%. In case of disagreement, the majority option was chosen.

## Data and Materials Availability.

Data and materials can be found at https://osf.io/zuehj/?view_only=29a2706c48cc4c9697a54596b26986f0 (57). Some study data available. (The materials for Experiment 5 cannot be publicly shared due to copyright restrictions, but will be available upon request.)
